# Oral Health Status in Patients with Inflammatory Bowel Diseases: A Systematic Review

**DOI:** 10.3390/ijerph182111521

**Published:** 2021-11-02

**Authors:** Kacper Nijakowski, Dawid Gruszczyński, Anna Surdacka

**Affiliations:** 1 Department of Conservative Dentistry and Endodontics, Poznan University of Medical Sciences, 60-812 Poznan, Poland; annasurd@ump.edu.pl; 2 Student’s Scientific Group in Department of Conservative Dentistry and Endodontics, Poznan University of Medical Sciences, 60-812 Poznan, Poland; dawid.j.gruszczynski@gmail.com

**Keywords:** inflammatory bowel disease, oral health, oral hygiene, dental caries, periodontal disease, Crohn’s disease, ulcerative colitis, systematic review

## Abstract

Inflammatory bowel diseases (IBD) are chronic disorders that affect the gastrointestinal tract, including the oral cavity. This systematic review was designed to answer the question “Is there a relationship between oral health status and inflammatory bowel diseases?”. Following the inclusion and exclusion criteria, fifteen studies were included (according to PRISMA statement guidelines). Due to their heterogeneity, only six articles about the prevalence of periodontal disease in IBD patients were included in the meta-analysis. Both Crohn’s disease (CD) and ulcerative colitis (UC) patients had an increased odds of periodontitis coincidence compared to the controls, more than 2- and 3-fold, respectively. Moreover, in most studies, patients with IBD were characterized by higher values of caries indices. In conclusion, despite the conducted systematic review, the risk of oral diseases in IBD patients cannot be clearly established due to the possible association of other factors, e.g., sociodemographic or environmental factors.

## 1. Introduction

Inflammatory bowel diseases (IBD) are chronic disorders that affect the gastrointestinal tract, including the oral cavity. Potential etiopathogenetic factors include genetic predisposition, immunological dysfunctions and environmental conditions [1]. Crohn’s disease (CD) and ulcerative colitis (UC) differ not so much in their clinical symptoms as in the extent of the inflammatory processes and their reflection in biochemical parameters of body fluids, e.g., saliva [2]. The course of the disease is associated with disturbances in the immune system, resulting in changes in proinflammatory cytokines and oxidative status markers [3]. Patients with IBD may have specific manifestations in the oral mucosa, such as cobblestoning, mucosal tags or deep linear ulcerations (in CD) and *pyostomatitis vegetans* (in UC) [4].

The most common dental problems include caries and periodontal disease. Carious lesions are formed due to changes occurring in the biofilm and thus disturbances in the neutral environment of the oral cavity, causing the demineralization of enamel [5]. Apart from the patient’s hygiene and dietary habits, other factors, such as medication and systemic diseases, that may affect saliva secretion are also important in the development of caries [6]. Additionally, periodontal diseases with alveolar bone destruction appear as a result of dysbiosis, often in patients with an impaired host response [7]. In the literature, factors increasing the risk of periodontal disease suggest, among others, systemic conditions such as diabetes, immunodeficiency, stress or obesity [8,9]. On the other hand, smoking can mask signs of bleeding on probing due to the contraction of the microvessels [10].

Both in the progression of IBD and periodontal disease, the key role is played by immunoinflammatory processes involving cytokines responsible for tissue destruction [11,12]. In addition, periopathogens may alter the composition of the intestinal microflora and exacerbate inflammatory processes, disrupting host defence [13,14]. In contrast, there is also a controversial thesis that poor oral hygiene may be associated with a reduced risk of developing IBD [15]. Based on our own observations, we found that the oral health status in IBD patients at our center has improved over the last decade [16]. Furthermore, other authors provide divergent information on the dental and periodontal status in this group of patients. Therefore, our systematic review was designed in order to answer the question “Is there a relationship between oral health status and inflammatory bowel diseases?”, formulated according to PICO (“Population”, “Intervention”, “Comparison” and “Outcome”).

## 2. Materials and Methods

### 2.1. Search Strategy and Data Extraction

A systematic review was conducted up to 1 October 2021, according to the Preferred Reporting Items for Systematic Reviews and Meta-Analyses (PRISMA) statement guidelines [17], using the databases PubMed, Scopus and Web of Science. The search formulas included:

- For PubMed: ((inflammatory bowel disease[MeSH Terms]) OR (crohn disease[MeSH Terms]) OR (ulcerative colitis[MeSH Terms])) AND ((oral health[MeSH Terms]) OR (caries, dental[MeSH Terms]) OR (periodontal disease[MeSH Terms]) OR (oral hygiene[MeSH Terms]))
- For Scopus: INDEXTERMS ((“inflammatory bowel disease” OR “Crohn disease” OR “ulcerative colitis”) AND (“oral health” OR “dental caries” OR “periodontal disease” OR “oral hygiene”))
- For Web of Science: KP = (inflammatory bowel disease OR Crohn disease OR ulcerative colitis) AND KP = (oral health OR dental caries OR periodontal disease OR oral hygiene).

Records were screened by the title, abstract and full text by 2 independent investigators. Studies included in this review matched all the predefined criteria according to PICOS (“Population”, “Intervention”, “Comparison”, “Outcomes”, and “Study design”), as shown in Table 1. A detailed search flowchart is presented in Section 3.

Because of the heterogeneity of the determined clinical indices, only 6 relatively homogeneous papers demonstrating the incidence of periodontal disease in IBD patients were included in the meta-analysis. The results of the meta-analysis were presented using forest plots.

### 2.2. Quality Assessment and Critical Appraisal for the Systematic Review of Included Studies

**Figure 1:**
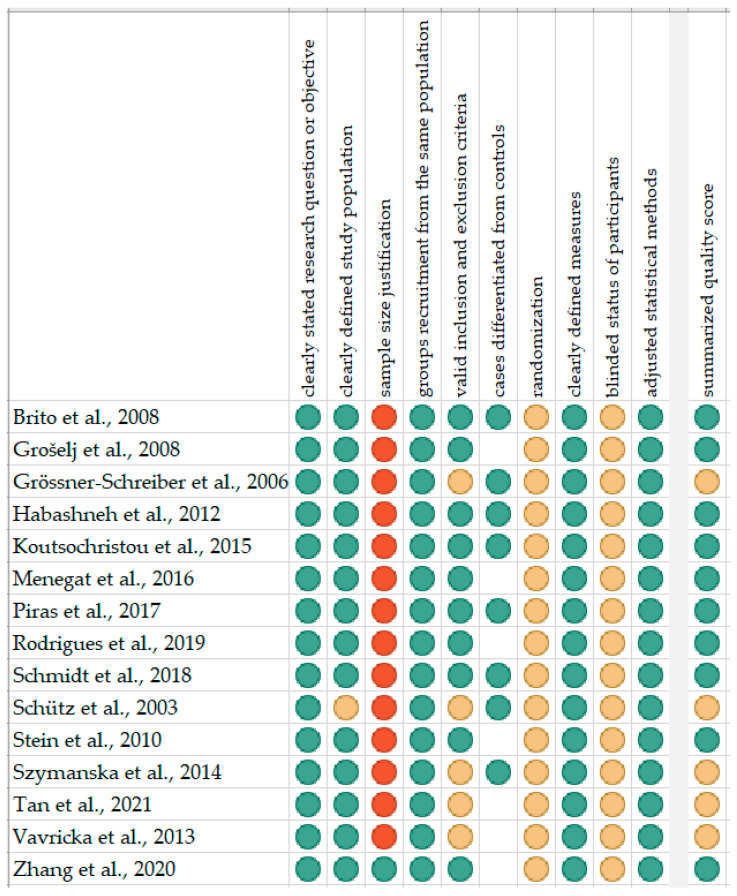
Quality assessment, including the main potential risk of bias (risk level: green—low, yellow—unspecified, red—high; quality score: green—good, yellow—intermediate, red—poor).

The risk of bias in each individual study was assessed according to the “Study Quality Assessment Tool” issued by the National Heart, Lung, and Blood Institute within the National Institute of Health [18]. These questionnaires were answered by 2 independent investigators, and any disagreements were resolved by discussion between them. The summarised quality assessment for every single study is reported in Figure 1. The most frequently encountered risks of bias were the absence of data regarding sample size justification (except for one study), randomization (all studies) and blinding (all studies). Critical appraisal was summarised by adding up the points for each criterion of potential risk (points: 1—low, 0,5—unspecified, 0—high). Ten studies (66.7%) were classified as having “good” quality (≥80% total score) and five (33.3%) as “intermediate” (≥60% total score).

The level of evidence was assessed using the classification of the Oxford Centre for Evidence-Based Medicine levels for diagnosis [19]. All of the included studies have the third or fourth level of evidence (in this 5-graded scale).

## 3. Results

**Figure 2:**
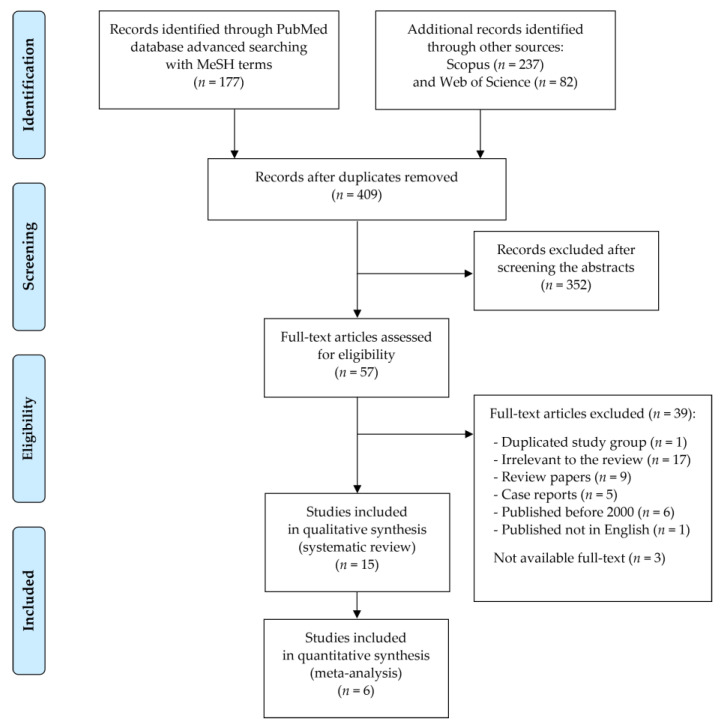
PRISMA flow diagram presenting search strategy.

In this systematic review, fifteen studies following the search criteria were included, and data were collected in eleven different countries from a total of 1748 participants (including 1104 patients with Crohn’s disease and 534 with ulcerative colitis and110 patients from one study without reported IBD forms). Figure 2 shows the detailed selection strategy of the articles. The inclusion and exclusion criteria are presented in Table 1 (in Section 2).

From each eligible study included in the present systematic review, data about its general characteristics, such as year of publication and setting, involved participants, pharmacological treatment prior to the study and smoking habits and assessed clinical indices were collected in Table 2. The majority of studies had complete information about determined parameters. Four of the included studies did not have a control group, and the others usually had healthy subjects with correctly matched demographic features to the study group. In the study by Menegat et al. [20], only IBD patients diagnosed with periodontal disease were included. Practically all the papers described the condition of the oral cavity, taking into account both dental and periodontal status. Only one study by Koutsochristou et al. [21] involved patients under the age of 18. Additionally, Table 3 presents the values of evaluated clinical indices and their reported statistical significance.

**Figure 3:**
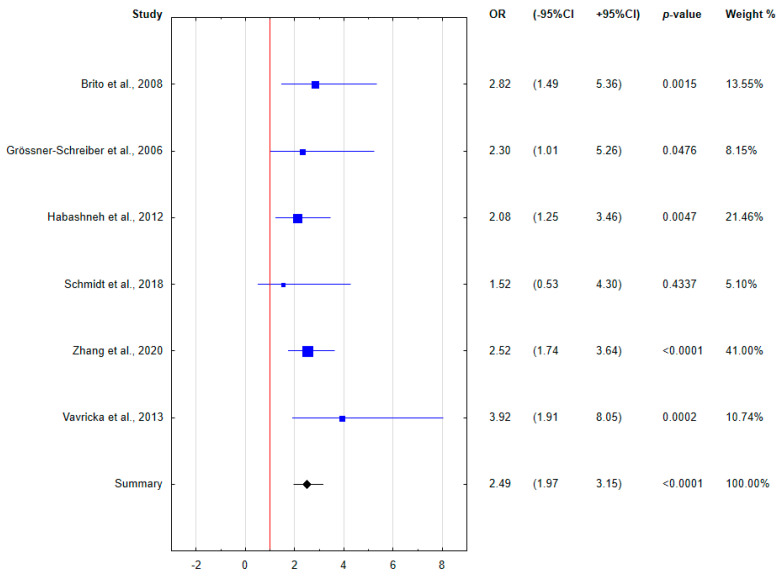
Forest plot presenting the odds for periodontal disease in IBD patients (OR, odds ratio; CI, confidence interval).

**Figure 4:**
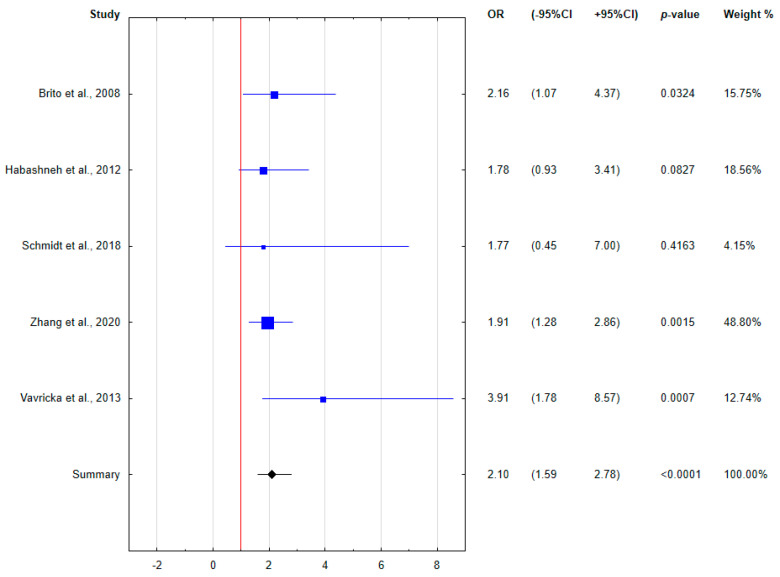
Forest plot presenting the odds for periodontal disease in CD patients (OR, odds ratio; CI, confidence interval).

**Figure 5:**
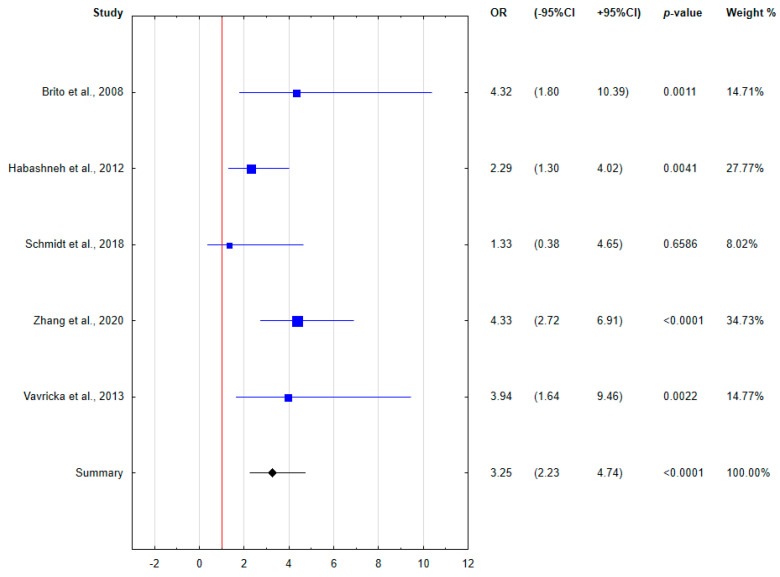
Forest plot presenting the odds for periodontal disease in UC patients (OR, odds ratio; CI, confidence interval).

The meta-analysis assessed the relationship between concurrent periodontal disease and IBD. The calculated odds ratios are presented in the forest plots (Figure 3, Figure 4 and Figure 5). Patients with IBD were found to be almost two and a half times more likely to have periodontal disease comorbidity. Taking into account the division into disease forms, both CD and UC patients were characterized by an increased odds of periodontal disease coincidence compared to healthy subjects, more than two- and three-fold, respectively. Based on the forest plots, the odds ratio was not significant for all subgroups in the study by Schmidt et al. [28] with the lowest weight, and was at the limit of significance only for CD patients in the study by Habashneh et al. [25].

## 4. Discussion

There are reports in the literature about the relationship between inflammatory bowel diseases and a predisposition to periodontal inflammatory diseases. In the study by Brito et al. [22], a higher prevalence of periodontal disease and caries was observed in patients with IBD, considering smoking as an important modifier of oral health. In the group of smokers, patients with UC manifested periodontal disease significantly more frequently and thus higher PPD values, and patients with CD had higher DMF-t values relative to healthy subjects. For nonsmokers, these relationships were reversed, with CD patients having significantly deeper periodontal pockets and UC patients having higher caries incidence. Similarly, the results of the study by Koutsochristou et al. [21] presented an increased incidence of caries and periodontal disease in children and adolescents with IBD, despite oral hygiene indicators comparable to the control group.

Moreover, Rodrigues et al. [27] observed the significantly increased prevalence of dental caries in UC patients. However, it was not influenced by their eating habits, such as the frequency of soft drinks, cakes, sweets and sugars between meals. Patients with the active form of the disease and a longer duration demonstrated higher levels of *Streptococcus mutans*, which seemed to be a manifestation of UC dysbiosis. These detected bacteria are mainly responsible for the caries development. Also, the study by Szymanska et al. [31] presents that CD patients after resective surgery had higher DMF-s scores and elevated counts of *Lactobacilli* and *Streptococcus mutans* in comparison to the control group. In contrast, Schütz et al. [29] noticed that in CD patients, caries prevalence was increased by a longer disease duration but depended on insufficient oral hygiene and intensified sugar consumption. Tan et al. [32] observed a significant increase in the DMF-t index only in the CD group but not in the UC group, while the periodontal status did not differ between IBD patients and healthy subjects.

Zhang et al. [34] compared the prevalence and severity of dental caries and periodontal disease in IBD and healthy subjects. Patients with CD and UC had significantly increased risks of caries and periodontitis compared to the control group. Habashneh et al. [25] also inferred a higher prevalence of periodontal disease of greater severity and extent in patients with IBD. In addition, deep oral mucosal ulcerations were significantly more common in the UC group. Furthermore, Vavricka et al. [33] suggested that predisposing factors for periodontal disease include an active form of CD with associated perianal lesions. Interestingly, Grošelj et al. [23] indicated that the selected parameters of oral health status could be used for predicting the clinical response in CD patients with infliximab administration. However, the prediction quality began to decrease after 2 months of anti-TNF therapy.

Menegat et al. [20] determined that the expression of selected cytokines was significantly increased in gingival tissue compared to the intestinal mucosa in IBD patients with periodontal disease. In the study by Schmidt et al. [28], IBD patients demonstrated more severe periodontitis with higher CAL compared to healthy subjects. Elevated aMMP−8 concentrations were correlated with periodontitis severity in only CD patients. Although the authors speculated changes in host immune response, the role of periodontal bacteria in the relation between periodontal diseases and IBD remained still unclear. In contrast, Stein et al. [30] suggested that CD patients had an increased prevalence of periodontal diseases. The colonization of *Campylobacter rectus* seems to be a causal pathogen for the periodontal manifestation in CD.

Surprisingly, Grössner-Schreiber et al. [24] observed no significant differences in caries incidence and periodontal status indices between IBD patients and healthy subjects, despite the determination of higher values of plaque indices in the study group. Similarly, in the study by Piras et al. [26], patients with IBD, especially those taking immunomodulators, demonstrated a higher prevalence of periapical lesions of larger sizes, despite the lack of differences in the caries indices in relation to the controls.

## 5. Conclusions

This systematic review suggests higher prevalence of dental caries and an increased risk of periodontal disease in IBD patients, especially those with ulcerative colitis. Therefore, the relationship between oral health status and inflammatory bowel diseases cannot be clearly defined due to confounders such as sociodemographic or environmental factors.

## Figures and Tables

**Table 1:** Inclusion and exclusion criteria according to the PICOS.

Parameter	Inclusion Criteria	Exclusion Criteria
Population	patients with IBD (Crohn’s disease and ulcerative colitis)—aged from 0 to 99 years, both sexes	patients with another bowel disease or autoimmune disease
Intervention	not applicable	
Comparison	not applicable	
Outcomes	determined clinical indices evaluating oral hygiene, as well as dental and periodontal status	determined only the presence of oral lesions
Study design	case-control, cohort and cross-sectional studies	literature reviews, case reports, expert opinion, letters to the editor, conference reports
	published after 2000	not published in English

**Table 2:** General characteristics of included studies.

Author, Year, Setting	IBD Patients (F/M)	Control Patients (F/M)	Pharmacological Treatment	Smoking Habits	Clinical Indices
Brito et al., 2008, Brazil [22]	CD: 99 (68/31); UC: 80 (47/33)	74 (50/24)	CD: Corticosteroids (*n* = 26), immunomodulators (*n* = 56), aminosalicylates (*n* = 60), anti-TNF (*n* = 7) and antibiotics (*n* = 8); UC: Corticosteroids (*n* = 15), immunomodulators (*n* = 19), aminosalicylates (*n* = 74), anti-TNF (*n* = 3) and antibiotics (*n* = 1)	CD: 12 smokers, 24 former smokers; UC: 7 smokers, 35 former smokers; Ctrl: 9 smokers, 8 former smokers	number of teeth, DMF-t, PCR, BOP, PPD, CAL
Grošelj et al., 2008, Slovenia [23]	CD: 14 (8/6)	-	Conventional treatment without response	NR	number of teeth, D-t, M-t, F-t, RCT teeth, PlI, GI, BOP, PPD, CAL
Grössner-Schreiber et al., 2006, Germany [24]	CD: 46; UC: 16	59 (sex-matched)	Corticosteroids (*n* = 20), immunosupressants (*n* = 24), aminosalicylates (*n* = 39), anti-TNF (*n* = 13) and antibiotics (*n* = 12)	CD: 24 smokers; UC: 1 smoker; IBD: 3 former smokers; Ctrl: 24 smokers; 6 former smokers	number of teeth, DMF-s, PCR, BOP, PPD
Habashneh et al., 2012, Jordan [25]	CD: 59 (26/33); UC: 101 (40/61)	100 (38/62)	NR	CD: 31 smokers, 5 former smokers; UC: 17 smokers, 29 former smokers; Ctrl: 49 smokers, 7 former smokers	PlI, GI, PPD, CAL, GR, BOP
Koutsochristou et al., 2015, Greece [21]	CD: 36 (18/18); UC: 19 (12/7)	55 (30/25)	Corticosteroids (*n* = 32), immunomodulators (*n* = 28), aminosalicylates (*n* = 50), anti-TNF (*n* = 9)	non-smokers	dmf-t, DMF-t, BOP, PCR
Menegat et al., 2016, Brazil [20]	CD: 18 (12/6); UC: 10 (7/3)	-	CD: Corticosteroids (*n* = 1), immunomodulators (*n* = 23), aminosalicylates (*n* = 21); UC: Corticosteroids (*n* = 1), immunomodulators (*n* = 9), aminosalicylates (*n* = 12)	CD: 2 smokers, 1 former smoker;UC: 3 former smokers	number of teeth, VPI, BOP, PPD, CAL
Piras et al., 2017, Italy [26]	110 (61/39)	110 (57/53)	Corticosteroids (*n* = 36), anti-TNF (*n* = 74)	NR	number of teeth, DMF-t
Rodrigues et al., 2019, Portugal [27]	UC: 30 (17/13)	-	Corticosteroids (*n* = 2), immunosupressants (*n* = 4), aminosalicylates (*n* = 13), anti-TNF (*n* = 7)	7 smokers	PlI, DMF-t
Schmidt et al., 2018, Germany [28]	CD: 29 (17/12); UC: 30 (17/13)	59 (34/25)	Corticosteroids (*n* = 5), immunomodulators (*n* = 26), aminosalicylates (*n* = 12), anti-TNF (*n* = 8)	CD: 14 smokers; Ctrl: 20 smokers	D-t, M-t, F-t, DMF-t, PBI, PPD, CAL
Schütz et al., 2003, Germany [29]	CD: 24 (14/10)	24 (12/12)	Corticosteroids (*n* = 11), immunosupressants (*n* = 2), aminosalicylates (*n* = 17), antibiotics (*n* = 1)	CD: 13 smokers; Ctrl: 12 smokers	DMF-t, API
Stein et al., 2010, Germany [30]	CD: 147 (77/70)	-	Corticosteroids (*n* = 62), immunosupressants (*n* = 70), aminosalicylates (*n* = 48)	55 smokers	missing teeth, PlI, GI, BOP, PPD, CAL
Szymanska et al., 2014, Sweden [31]	CD: 150 (73/77)	75 (45/30)	NR(71 after resective surgery)	CD: 44 smokers; Ctrl: 6 smokers	D-t, M-t, F-t, DMF-t, D-s, M-s, F-s, DMF-s, VPI
Tan et al., 2021, Netherlands [32]	CD: 148 (88/60); UC: 80 (44/36)	229 (133/96)	Corticosteroids (*n* = 36), immunosupressants (*n* = 25), aminosalicylates (*n* = 59), anti-TNF (*n* = 27)	IBD: 53 smokers; Ctrl: 72 smokers	DMF-t
Vavricka et al., 2013, Switzerland [33]	CD: 69 (32/37); UC: 44 (16/28)	113 (55/58)	CD: Corticosteroids (*n* = 12), immunosupressants (*n* = 17), aminosalicylates (*n* = 8), anti-TNF (*n* = 36); UC: Corticosteroids (*n* = 12), immunosupressants (*n* = 19), aminosalicylates (*n* = 29), anti-TNF (*n* = 9)	CD: 21 smokers, 3 former smokers; UC: 2 smokers, 19 former smokers; Ctrl: 21 smokers, 21 former smokers	number of teeth, DMF-t, BOP, LA-PPD, PBI
Zhang et al., 2020, China [34]	CD: 265 (95/170); UC: 124 (49/75)	265 (115/150)	CD: Corticosteroids (*n* = 10), immunosupressants (*n* = 106), aminosalicylates (*n* = 26), anti-TNF (*n* = 106); UC: Corticosteroids (*n* = 18), immunosupressants (*n* = 27), aminosalicylates (*n* = 68), anti-TNF (*n* = 7)	CD: 21 smokers, 36 former smokers; UC: 14 smokers, 23 former smokers; Ctrl: 22 smokers, 17 former smokers	D-t, M-t, F-t, DMF-t, D-s, M-s, F-s, DMF-s, PlI, GI, PPD, CAL, BOP, CI, GR

Legend: CD, Crohn’s disease; UC, ulcerative colitis; IBD, inflammatory bowel disease; F, females; M, males; Ctrl, control group; NR, not reported; TNF, tumour necrosis factor; D, decayed; M, missing; F, filled; t, tooth; s, surface; PlI, plaque index (Silness&Löe); API, approximal plaque index; GI, gingival index; BOP, bleeding on probing; PPD, periodontal probing depth; CAL, clinical attachment level; GR, gingival recession; PCR, plaque control record (O’Leary); VPI, visual plaque index; LA-PPD, loss of attachment at sites with maximal periodontal pocket depth; PBI, papilla bleeding index; CI, calculus index.

**Table 3:** Statistical significance for dental and periodontal indices in IBD patients.

Study	Clinical Indices	Mean ± SD/Median (Q1–Q3)		*p*-Value		
		CD	UC	CD vs. UC	IBD vs. ctrl	
					CD vs. ctrl	UC vs. ctrl
Brito et al., 2008 [22]	number of teeth	24.0 (9.0)	22.0 (10.0)	0.086 ^#^	0.133	0.002 *
	DMF-t	15.1 ± 7.2	16.4 ± 6.6	0.229	0.018 *	<0.0001 *
	PCR	38.2 (47.4)	53.7 (60.4)	0.239	0.017 *	0.479
	BOP	19.6 (20.5)	21.5 (21.9)	0.308	0.038 *	0.265
	PPD	2.3 (1.3)	2.3 (0.4)	0.941	<0.0001 *	<0.0001 *
	CAL	0.9 (0.9)	1.3 (1.4)	0.005 *	0.576	0.004 *
Grošelj et al., 2008 [23]	number of teeth	25.1 ± 5.7	-	-	-	-
	D-t	5.7 ± 3.8	-	-	-	-
	M-t	5.9 ± 6.3	-	-	-	-
	F-t	8.7 ± 4.5	-	-	-	-
	RCT teeth	1.1 ± 1.3	-	-	-	-
	PlI	0.7 ± 0.4	-	-	-	-
	GI	0.7 ± 0.4	-	-	-	-
	BOP	0.2 ± 0.1	-	-	-	-
	PPD	1.7 ± 0.4	-	-	-	-
	CAL	1.8 ± 0.8	-	-	-	-
Grössner-Schreiber et al., 2006 [24]	number of teeth	27 (24–28)		-	0.148	
	DMF-s	46.0 (32.8–66.3)		-	0.212	
	PCR	33.3 (16.7–62.5)		-	0.032 *	
	BOP	16.7 (8.3–30.8)		-	0.958	
	PPD	2.08 (1.82–2.34)		-	0.014 *	
Habashneh et al., 2012 [25]	PlI	2.32 ± 0.70	2.51 ± 0.52	NS	<0.05 *	<0.05 *
	GI	2.32 ± 0.70	2.41 ± 0.65	NS	<0.05 *	<0.05 *
	PPD	1.29 ± 0.47	1.51 ± 0.47	<0.05 *	NS	<0.05 *
	CAL	1.95 ± 0.98	2.36 ± 1.13	<0.05 *	NS	<0.05 *
	GR	0.53 ± 0.55	0.86 ± 0.72	<0.05 *	NS	<0.05 *
	BOP	10.84 ± 16.20	10.20 ± 14.25	NS	<0.05 *	<0.05 *
Koutsochristou et al., 2015 [21]	dmf-t	2.95 ± 1.87		-	<0.001 *	
	DMF-t	5.81 ± 2.05		-	<0.001 *	
	BOP	40.24 ± 13.81		-	<0.001 *	
	PCR	42.29 ± 14.03		-	NS	
Menegat et al., 2016 [20]	number of teeth	22.00 (12.25)	16.00 (13.00)	0.865	-	-
	VPI	67.50 (27.75)	66.73 (43.60)	0.474	-	-
	BOP	66.46 (37.16)	78.98 (48.79)	0.692	-	-
	PPD	2.86 (0.52)	2.71 (0.56)	0.340	-	-
	CAL	2.88 (1.11)	2.97 (0.70)	0.177	-	-
Piras et al., 2017 [26]	number of teeth	25 ± 6.1		-	0.0004 *	
	DMF-t	9.1 ± 4.6		-	0.93	
Rodrigues et al., 2019 [27]	PlI	-	0.849 ± 0.638	-	-	-
	DMF-t	-	16.17 ± 6.428	-	-	-
Schmidt et al., 2018 [28]	D-t	1.07 ± 2.6	0.40 ± 0.8	-	<0.01 *	
	M-t	4.45 ± 4.7	4.27 ± 5.1	-	0.19	
	F-t	10.45 ± 4.1	12.40 ± 4.8	-	0.68	
	DMF-t	15.97 ± 6.3	17.07 ± 5.6	-	0.23	
	PBI	1.43 ± 0.9	1.09 ± 0.8	-	0.81	
	PPD	2.49 ± 1.1	2.26 ± 1.1	-	<0.01 *	
	CAL	3.27 ± 1.1	3.39 ± 1.5	-	<0.01 *	
Schütz et al., 2003 [29]	DMF-t	dd <3 years: 9.5 ± 4.3; >3 years: 15.6 ± 5.7	-	-	-	-
	API	85.5 ± 23.6	-	-	<0.001 *	-
Stein et al., 2010 [30]	missing teeth	6.1 ± 3.7	-	-	-	-
	PlI	1.2 ± 0.6	-	-	-	-
	GI	1.2 ± 0.6	-	-	-	-
	BOP	23.9 ± 7.8	-	-	-	-
	PPD	3.6 ± 0.8	-	-	-	-
	CAL	3.8 ± 1.0	-	-	-	-
Szymanska et al., 2014 [31]	D-t	with/without RS: 2.2 ± 3.2; 1.8 ± 2.9	-	-	NS	-
	M-t	2.7 ± 4.1; 1.8 ± 2.9	-	-	NS	-
	F-t	10.6 ± 6.4; 8.0 ± 5.4	-	-	NS	-
	DMF-t	15.5 ± 8.3; 11.2 ± 7.1	-	-	NS	-
	D-s	3.6 ± 7.6; 2.7 ± 5.9	-	-	NS	-
	M-s	13.3 ± 19.9; 8.9 ± 13.7	-	-	NS	-
	F-s	33.7 ± 24.5; 22.6 ± 19.1	-	-	NS	-
	DMF-s	50.7 ± 36.2; 33.1 ± 28.6	-	-	0.014 *	-
	VPI	53.7 ± 29.2; 45.3 ± 25.9	-	-	0.001 *	-
Tan et al., 2021 [32]	DMF-t	14.6 ± 8.0	13.8 ± 7.5	-	0.002 *	0.643
Vavricka et al., 2013 [33]	number of teeth	28 (27–30)	28 (26–31)	-	0.10	0.80
	DMF-t	7 (2–12.75)	7 (3–13)	-	0.16	0.07
	PBI	0 (0–0.5)	0 (0–0.37)	-	<0.001 *	0.01 *
	LA-PPD	4 (3–5)	4 (3–5)	-	0.06^#^	0.05^#^
	BOP	0 (0–0)	0 (0–0)	-	0.004 *	0.38
Zhang et al., 2020 [34]	D-t	3 (1–6)	2 (1–5)	0.330	<0.001 *	<0.001 *
	M-t	0 (0–1)	0 (0–1)	0.300	<0.002 *	<0.002 *
	F-t	0 (0–2)	0 (0–1)	0.716	0.724	1.000
	DMF-t	5 (2–7)	4 (2–7)	0.711	<0.001 *	<0.001 *
	D-s	5 (2–9)	4 (1–8)	0.773	<0.001 *	<0.001 *
	M-s	0 (0–5)	0 (0–5)	0.280	<0.001 *	0.061^#^
	F-s	0 (0–3)	0 (0–2)	0.126	0.126	1.000
	DMF-s	7 (3–16)	6 (2–15)	0.154	<0.001 *	<0.001 *
	PlI	0.68 (0.47–0.94)	0.70 (0.54–0.98)	0.885	<0.001 *	<0.001 *
	GI	0.39 (0.22–0.61)	0.47 (0.27–0.72)	0.183	<0.001 *	<0.001 *
	PPD	1.89 (1.68–2.22)	2.10 (1.83–2.40)	0.902	<0.001 *	<0.001 *
	CAL	0.01 (0.00–0.17)	0.14 (0.01–0.38)	1.000	<0.001 *	<0.001 *
	BOP	14.81 (7.69–28.57)	22.77 (10.71–33.33)	0.279	<0.001 *	<0.001 *
	CI	53.57 (39.29–63.16)	58.01 (44.22–67.86)	0.847	<0.001 *	<0.001 *
	GR	0.00 (0.00–0.01)	0.00 (0.00–0.09)	1.000	0.007 *	0.173

Legend: CD, Crohn’s disease; UC, ulcerative colitis; IBD, inflammatory bowel disease; ctrl, control group; -, not applicable; D, decayed; M, missing; F, filled; t, tooth; s, surface; PlI, plaque index (Silness&Löe); API, approximal plaque index; GI, gingival index; BOP, bleeding on probing; PPD, periodontal probing depth; CAL, clinical attachment level; GR, gingival recession; PCR, plaque control record (O’Leary); VPI, visual plaque index; LA-PPD, loss of attachment at sites with maximal periodontal pocket depth; PBI, papilla bleeding index; CI, calculus index; dd, disease duration; RS, resective surgery; NS, not significant without reported *p*-value; *, statistical significance for *p*-value < 0.05; ^#^, suggestive *p*-value between 0.05 and 0.1.

## Data Availability

Data are available on request from the corresponding author.
